# Establishment of a monoclonal antibody PMab-225 against alpaca podoplanin for immunohistochemical analyses

**DOI:** 10.1016/j.bbrep.2019.100633

**Published:** 2019-04-05

**Authors:** Yukinari Kato, Yoshikazu Furusawa, Shinji Yamada, Shunsuke Itai, Junko Takei, Masato Sano, Mika K. Kaneko

**Affiliations:** a New Industry Creation Hatchery Center, Tohoku University, 2-1 Seiryo-machi, Aoba-ku, Sendai, Miyagi, 980-8575, Japan; b Department of Antibody Drug Development, Tohoku University Graduate School of Medicine, 2-1 Seiryo-machi, Aoba-ku, Sendai, Miyagi, 980-8575, Japan

**Keywords:** Alpaca podoplanin, PDPN, PMab-225, CBIS, Cell-Based Immunization and Screening, CHO, Chinese hamster ovary, CLEC-2, C-type lectin-like receptor-2, DAB, 3,3′-diaminobenzidine tetrahydrochloride, aPDPN, alpaca podoplanin, hPDPN, human podoplanin, mAb, monoclonal antibody, PBS, phosphate-buffered saline, PDPN, podoplanin, PVDF, polyvinylidene difluoride, SDS, sodium dodecyl sulfate

## Abstract

Podoplanin (PDPN) is known as a lymphatic endothelial cell marker. Monoclonal antibodies (mAbs) against human, mouse, rat, rabbit, dog, cat, bovine, pig, and horse PDPN have been established in our previous studies. However, mAbs against alpaca PDPN (aPDPN), required for immunohistochemical analysis, remain to be developed. In the present study, we employed the Cell-Based Immunization and Screening (CBIS) method for producing *anti*-aPDPN mAbs. We immunized mice with aPDPN-overexpressing Chinese hamster ovary (CHO)-K1 cells (CHO/aPDPN), and hybridomas producing mAbs against aPDPN were screened using flow cytometry. One of the mAbs, PMab-225 (IgG_2b_, kappa), specifically detected CHO/aPDPN cells via flow cytometry and recognized the aPDPN protein on Western blotting. Further, PMab-225 strongly stained lung type I alveolar cells, colon lymphatic endothelial cells, and kidney podocytes via immunohistochemistry. These findings demonstrate that PMab-225 antibody is useful to investigate the function of aPDPN via different techniques.

## Highlights

• PDPN is known as a specific lymphatic endothelial cell (LEC) marker.
• Sensitive and specific PMab-225 mAb against alpaca PDPN was produced.
• PMab-225 strongly reacted with alpaca PDPN in flow cytometry.
• PMab-225 is useful for IHC using paraffin-embedded cell sections.

## 1 Introduction

In many studies, alpaca (*lama pacos*) has been used for production of antigen-specific single domain antibodies (nanobodies) [[1], [2], [3]]. In contrast, membrane proteins of alpaca have not been investigated due to the lack of specific antibodies. The type I transmembrane glycoprotein, podoplanin (PDPN)/T1alpha/Aggrus, is expressed in normal tissues, including type I lung alveolar cells, renal podocytes, and lymphatic endothelial cells [[4], [5], [6]]. The interaction between PDPN on lymphatic endothelial cells and C-type lectin-like receptor-2 (CLEC-2) on platelets facilitates embryonic blood/lymphatic vessel separation [4,[6], [7], [8], [9], [10], [11], [12], [13]]. The expression of human PDPN (hPDPN) has been reported in several malignant tumors, including malignant brain tumors [[14], [15], [16], [17]], malignant mesotheliomas [18,19], oral squamous cell carcinomas [20], esophageal cancers [21], lung cancers [22], osteosarcomas [[23], [24], [25]], chondrosarcomas [24], and testicular tumors [26]. The expression of hPDPN is associated with malignant progression and cancer metastasis [9,14,27].

We have developed monoclonal antibodies (mAbs) against human [28], mouse [28], rat [29], rabbit [30], dog [31], cat [32], bovine [33], pig [34], and horse [35] PDPNs. However, mAbs against alpaca PDPN (aPDPN), useful for immunohistochemical analysis, remain to be developed. Sensitive and specific mAbs against aPDPN are necessary to investigate the expression and function of aPDPN. In the present study, we immunized mice with CHO/aPDPN cells and established hybridomas to produce mAbs against aPDPN.

## 2 Materials and methods

### 2.1 Cell lines

CHO-K1 and P3X63Ag8U.1 (P3U1) cells were obtained from the American Type Culture Collection (ATCC, Manassas, VA, USA). The coding sequence of aPDPN bearing an *N*-terminal RAP16 tag (RAP16-aPDPN) was inserted into a pCAG-Neo vector (FUJIFILM Wako Pure Chemical Corporation, Osaka, Japan). The RAP16 tag comprises 16 amino acids (GPGDDMVNPGLEDRIE). CHO-K1 cells were transfected with pCAG-Neo/RAP16-aPDPN using Lipofectamine LTX with Plus Reagent (Thermo Fisher Scientific Inc., Waltham, MA, USA). Stable transfectants were selected by limiting dilution and cultivating in a medium containing 0.5 mg/mL of G418 (Nacalai Tesque, Inc., Kyoto, Japan).

P3U1, CHO-K1, and CHO/aPDPN cells were cultured in Roswell Park Memorial Institute (RPMI) 1640 medium (Nacalai Tesque, Inc.). All the media were supplemented with 10% heat-inactivated fetal bovine serum (Thermo Fisher Scientific Inc.), 100 units/mL of penicillin, 100 μg/mL of streptomycin, and 25 μg/mL of amphotericin B (Nacalai Tesque, Inc.). Cells were grown at 37 °C in a humidified environment with an atmosphere of 5% CO_2_ and 95% air.

### 2.2 Hybridoma production

Female BALB/c mice (6 weeks old) were purchased from CLEA Japan (Tokyo, Japan). Animals were housed under specific pathogen-free conditions. The Animal Care and Use Committee of Tohoku University approved all the animal experiments. Two BALB/c mice were immunized with CHO/aPDPN cells (1 × 10^8^) intraperitoneally (i.p.) administered together with Imject Alum (Thermo Fisher Scientific Inc.). The procedure included three additional immunizations, followed by a final booster injection administered i.p. two days prior to the harvest of spleen cells, amounting to a total of five immunizations. These spleen cells were subsequently fused with P3U1 cells using PEG1500 (Roche Diagnostics, Indianapolis, IN, USA), and the hybridomas were grown in RPMI medium supplemented with hypoxanthine, aminopterin, and thymidine for selection (Thermo Fisher Scientific Inc.). The cultured supernatants were screened using flow cytometry.

### 2.3 Flow cytometry

The cells were harvested following brief exposure to 0.25% trypsin/1 mM ethylenediaminetetraacetic acid (EDTA; Nacalai Tesque, Inc.), washed with 0.1% bovine serum albumin (BSA)/phosphate-buffered saline (PBS), and treated with primary mAbs for 30 min at 4 °C. Thereafter, the cells were treated with Alexa Fluor 488-conjugated anti-mouse IgG (1:2000; Cell Signaling Technology, Inc., Danvers, MA, USA). Fluorescence data were collected using a SA3800 Cell Analyzer (Sony Corp., Tokyo, Japan).

### 2.4 Determination of binding affinity using flow cytometry

CHO/aPDPN was suspended in 100 μL of serially diluted PMab-225, followed by the addition of Alexa Fluor 488-conjugated anti-mouse IgG (1:200; Cell Signaling Technology, Inc.). Fluorescence data were collected using EC800 Cell Analyzer (Sony Corp.). The dissociation constant (*K*_D_) was obtained by fitting the binding isotherms to built-in one-site binding models in GraphPad PRISM 6 (GraphPad Software, Inc., La Jolla, CA, USA).

### 2.5 Western blotting

Cell lysates (10 μg) were boiled in sodium dodecyl sulfate sample buffer (Nacalai Tesque, Inc.). The proteins were subjected to electrophoresis on 5%–20% polyacrylamide gels (FUJIFILM Wako Pure Chemical Corporation) and subsequently transferred onto a polyvinylidene difluoride (PVDF) membrane (Merck KGaA, Darmstadt, Germany). After blocking with 4% skim milk (Nacalai Tesque, Inc.), each membrane was incubated with primary mouse mAbs, such as 1 μg/mL of PMab-225, anti-RAP16 tag (PMab-2), or *anti*-*β*-actin (AC-15; Sigma-Aldrich Corp., St. Louis, MO, USA), and subsequently with peroxidase-conjugated anti-mouse IgG (1:1000; Agilent Technologies, Santa Clara, CA, USA). Bands were visualized using ImmunoStar LD (FUJIFILM Wako Pure Chemical Corporation) using a Sayaca-Imager (DRC Co. Ltd., Tokyo, Japan).

### 2.6 Immunohistochemical analyses

Normal alpaca tissues were collected after autopsy at Hokkaido University, fixed in 10% neutral-buffered formalin [36], and routinely processed to make paraffin-embedded tissue sections. Histological sections of 4 μm thickness were directly autoclaved in citrate buffer (pH 6.0; Nichirei Biosciences, Inc., Tokyo, Japan) or EnVision FLEX Target Retrieval Solution High pH (Agilent Technologies Inc.) for 20 min. These tissue sections were blocked using SuperBlock T20 (PBS) Blocking Buffer (Thermo Fisher Scientific Inc.), incubated with PMab-225 (1 μg/mL or 5 μg/mL) for 1 h at room temperature, and treated using an Envision + Kit (Agilent Technologies Inc.) for 30 min. Color was developed using 3,3′-diaminobenzidine tetrahydrochloride (Agilent Technologies Inc.) for 2 min, and counterstaining was performed using hematoxylin (FUJIFILM Wako Pure Chemical Corporation).

## 3 Results

**Fig. 1:**
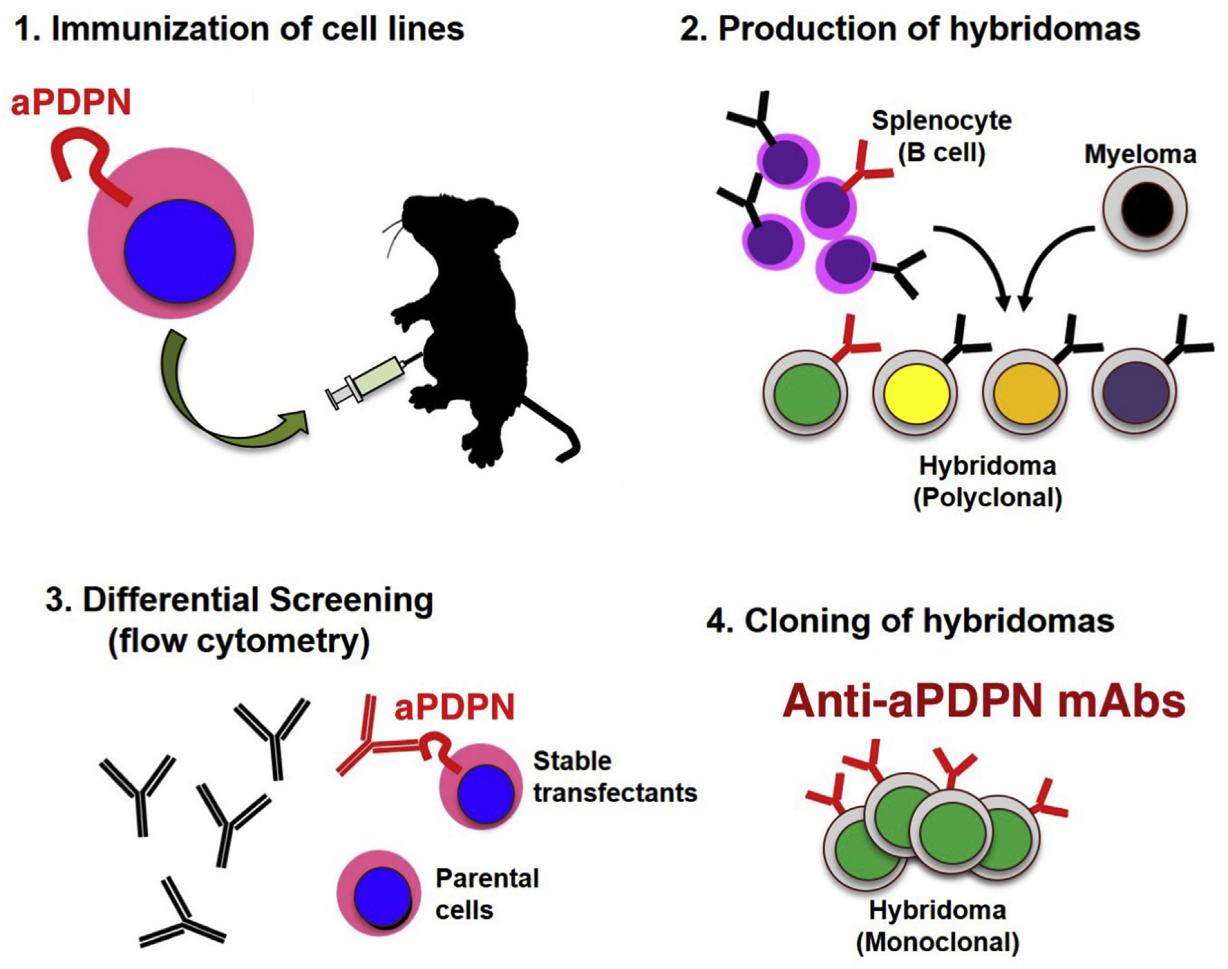
**Schematic illustration of the Cell-Based Immunization and Screening (CBIS) method.** Stable transfectants expressing the protein of interest are used as immunogens with no purification procedure. The selection of hybridomas secreting specific mAbs is performed by flow cytometry using parental and transfectant cells.

In this study, two mice were immunized with CHO/aPDPN cells (Fig. 1). Developed hybridomas were seeded into 96-well plates and cultivated for 8 days (first mouse) or 9 days (second mouse). Wells positive for CHO/aPDPN and negative for CHO-K1 were selected using flow cytometry. Screening identified strong signals against CHO/aPDPN cells and weak or no signals against CHO-K1 cells in 83 of 960 wells (8.6%). Of these 83 wells, two hybridomas were developed. One of these two clones, PMab-225 (IgG_2b_, kappa), was selected for immunohistochemistry against alpaca tissues.

**Fig. 2:**
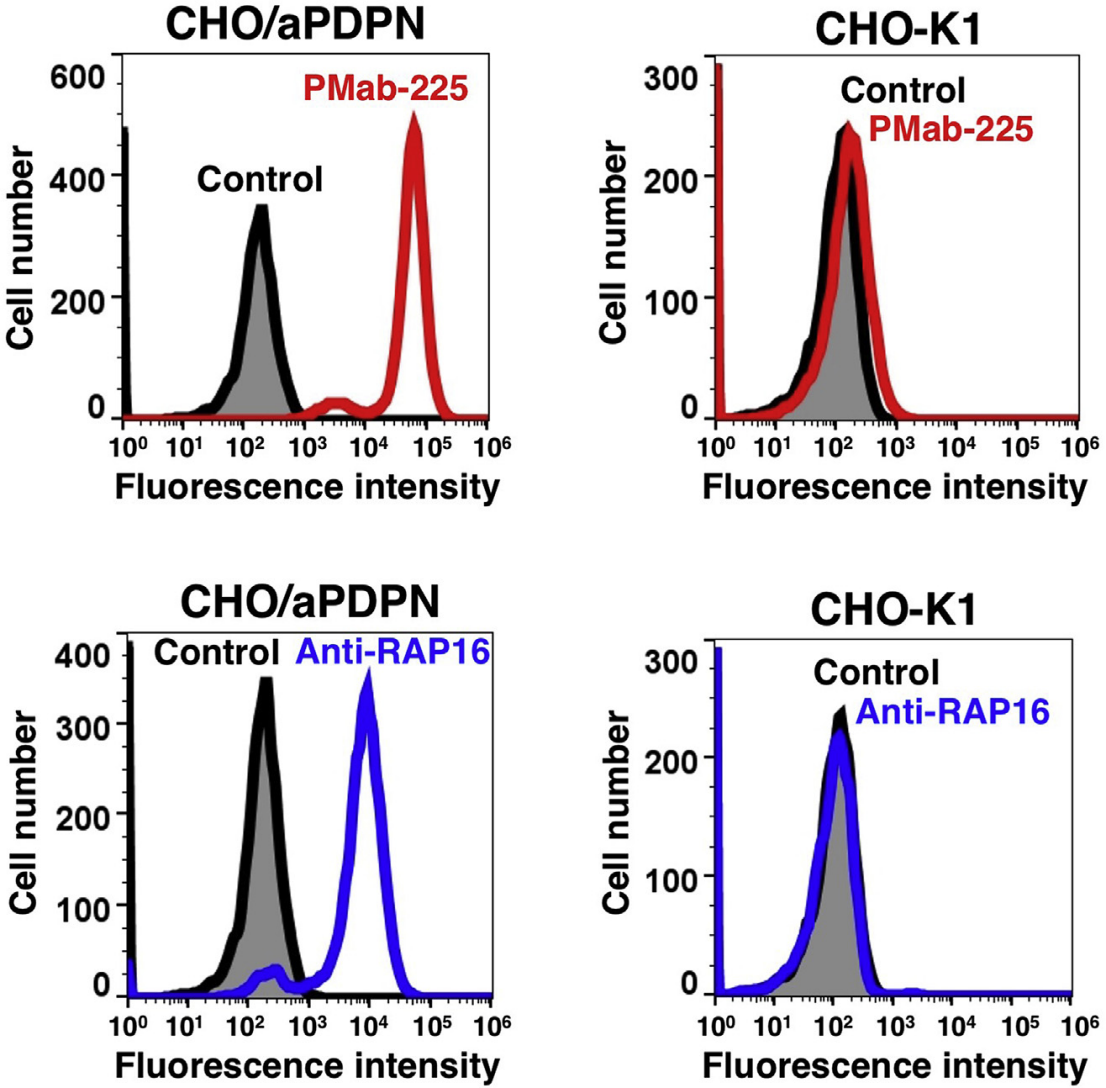
**Detection of aPDPN via flow cytometry using PMab-225.** CHO/aPDPN CHO-K1 cells were treated with 10 μg/mL of PMab-225 (red line) or 1 μg/mL of anti-RAP16 tag (PMab-2; blue line) or 0.1% BSA in PBS (gray) for 30 min, followed by incubation with secondary antibodies.

PMab-225 recognized CHO/aPDPN but showed no reaction with CHO-K1, as assessed using flow cytometry (Fig. 2). Additionally, a kinetic analysis performed using flow cytometry assessed the interaction of PMab-225 with CHO/aPDPN. *K*_D_ of PMab-225 for CHO/aPDPN cells was determined to be 2.4 × 10^−9^, indicating high affinity for CHO/aPDPN cells.

**Fig. 3:**
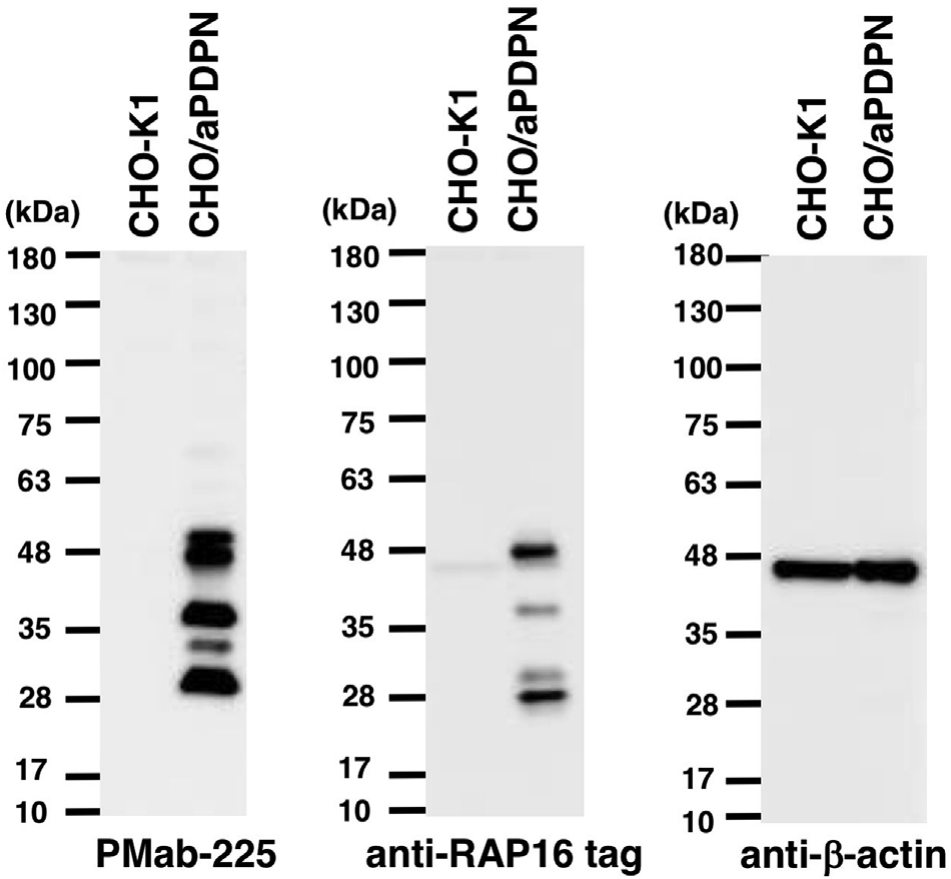
**Detection of aPDPN via Western blotting.** Cell lysates of CHO-K1 and CHO/aPDPN (10 μg) were electrophoresed and transferred onto PVDF membranes. The membranes were incubated with l μg/mL of PMab-225, anti-RAP16 tag (PMab-2), or *anti*-*β*-actin and subsequently with peroxidase-conjugated anti-mouse IgG.

Western blotting performed using PMab-225 (Fig. 3) demonstrated that PMab-225 detects aPDPN in CHO/aPDPN cells. PMab-2, an anti-RAP16 tag mAb, also detected aPDPN bands in CHO/aPDPN cells. Several bands were obtained that might represent highly glycosylated forms.

**Fig. 4:**
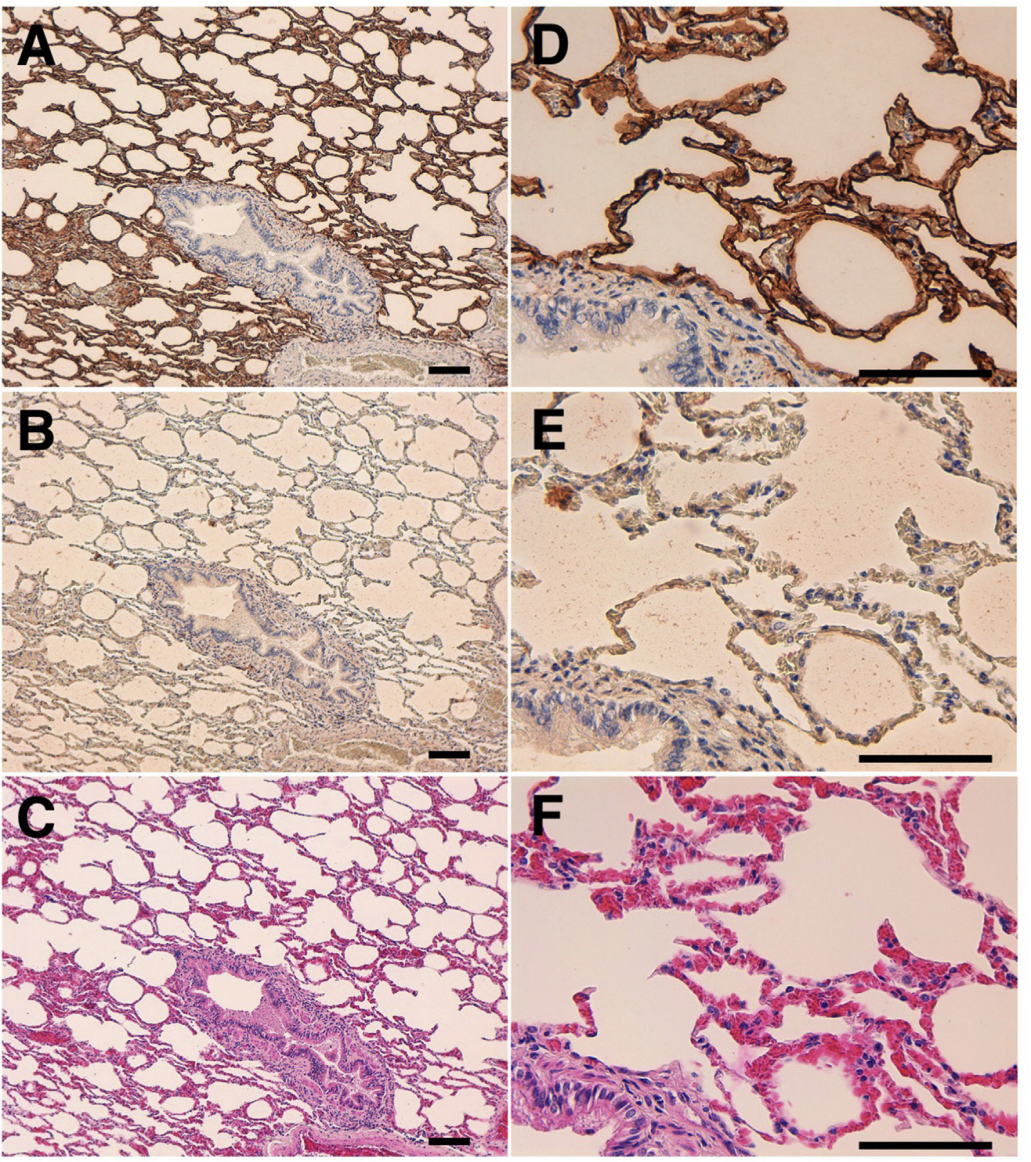
**Immunohistochemical analyses against alpaca lung.** Tissue sections of alpaca lung were directly autoclaved in citrate buffer and incubated with 1 μg/mL of PMab-225 (A, D) or with blocking buffer (B, E). Type I alveolar cells were stained. (C, F) Hematoxylin and eosin staining. Scale bar = 100 μm.

**Fig. 5:**
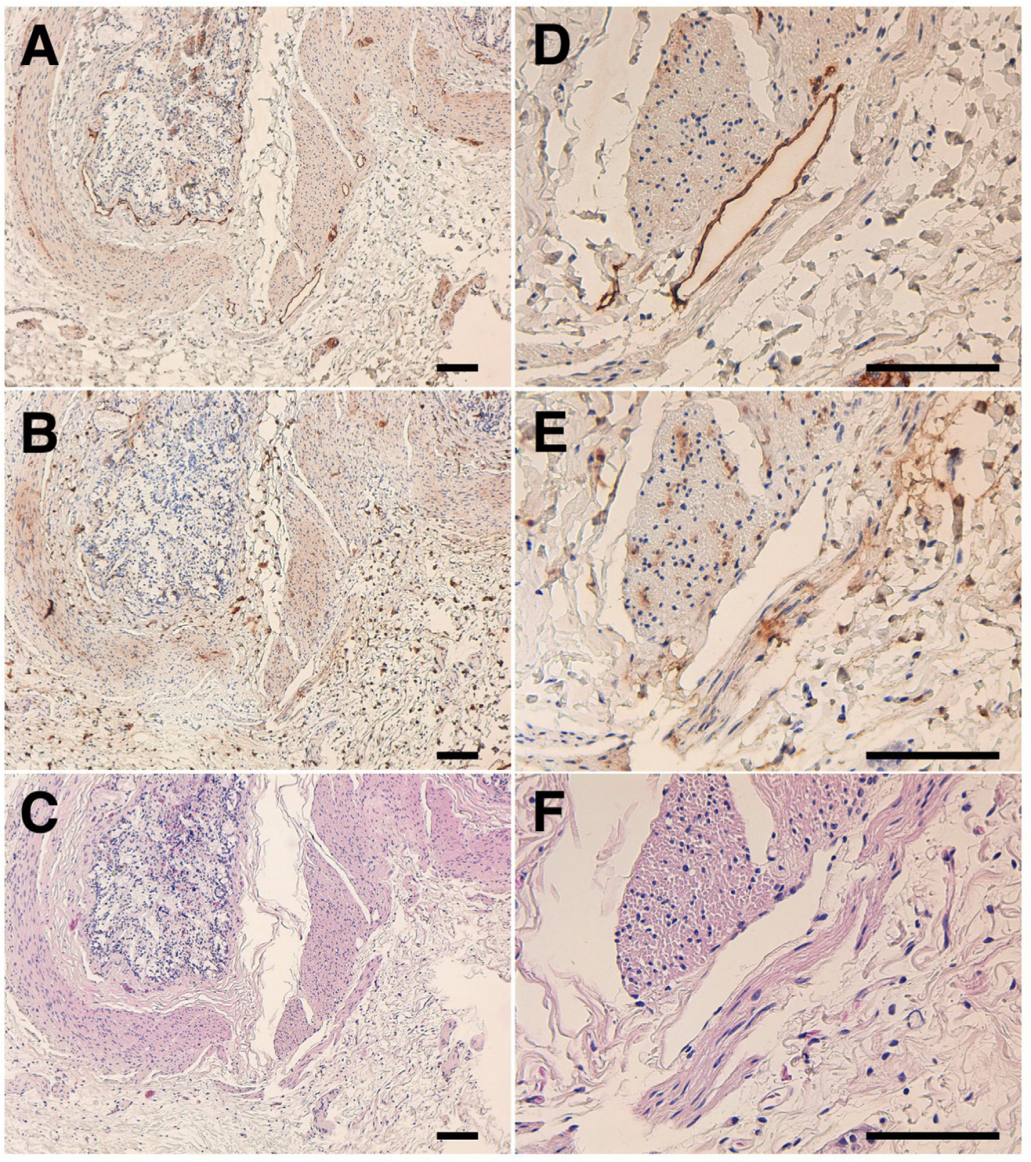
**Immunohistochemical analyses against alpaca colon.** Tissue sections of alpaca colon were directly autoclaved in citrate buffer and incubated with 1 μg/mL of PMab-225 (A, D) or with blocking buffer (B, E). Lymphatic endothelial cells were stained. (C, F) Hematoxylin and eosin staining. Scale bar = 100 μm.

**Fig. 6:**
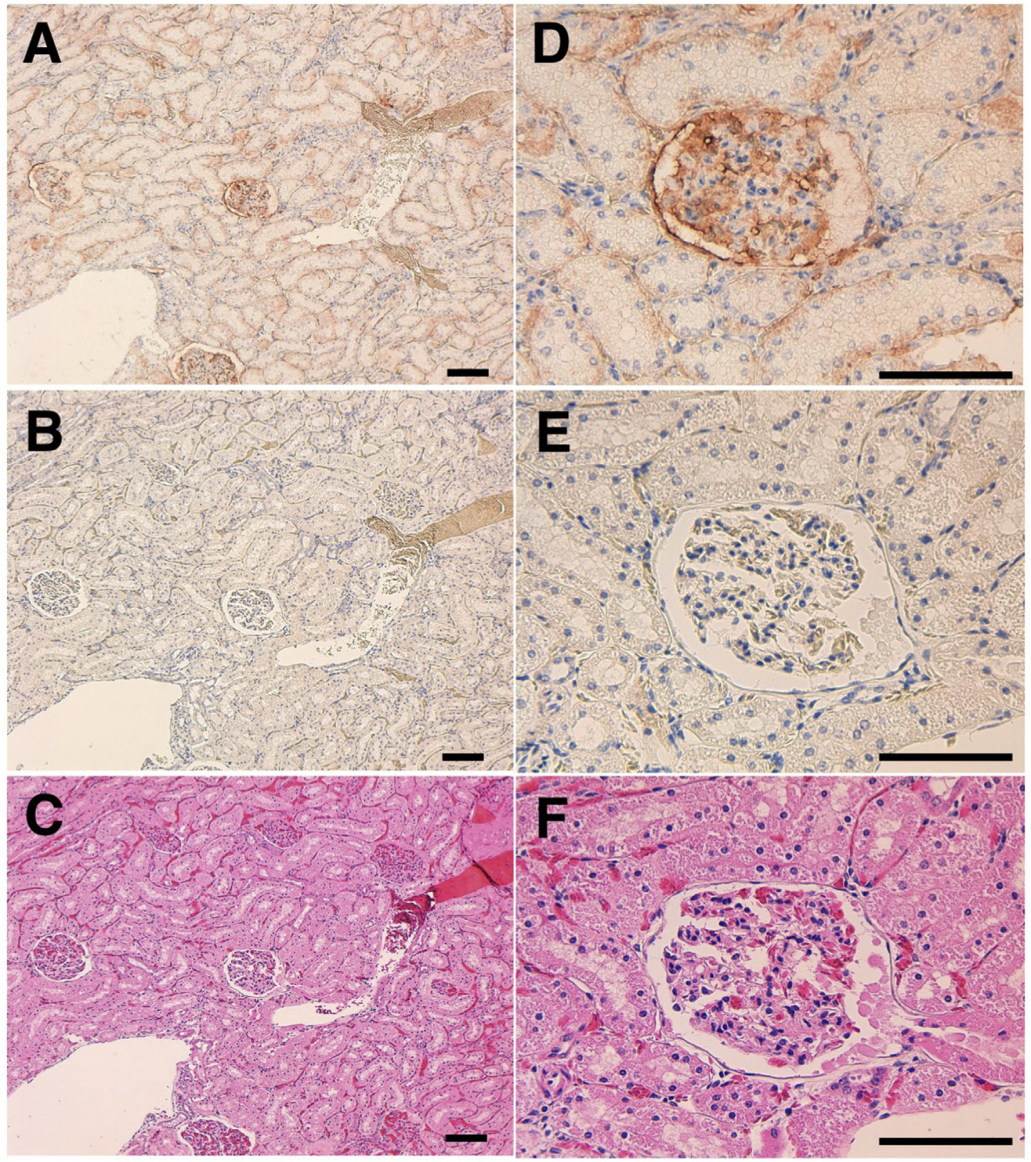
**Immunohistochemical analyses against alpaca kidney.** Tissue sections of alpaca kidney were directly autoclaved in EnVision FLEX Target Retrieval Solution High pH and incubated with 5 μg/mL of PMab-225 (A, D) or with blocking buffer (B, E). Podocytes and Bowman's capsule were stained (C, F) Hematoxylin and eosin staining. Scale bar = 100 μm.

The immunohistochemical analyses using antigen retrieval with citrate buffer (pH 6.0) revealed that PMab-225 strongly stained type I alveolar cells in the alpaca lung (Fig. 4) and lymphatic endothelial cells in alpaca colon tissues (Fig. 5). Podocytes and Bowman's capsule of alpaca kidney were stained using antigen retrieval with EnVision FLEX Target Retrieval Solution High pH (Fig. 6). These results indicate that PMab-225 will be useful to elucidate the pathophysiological functions of aPDPN in alpaca tissues in the future.

## 4 Discussion

In our previous studies, we established a cancer-specific monoclonal antibody (CasMab) technology to produce CasMabs, such as LpMab-2 and LpMab-23 against hPDPN, in several studies [17,37]. Those CasMabs against hPDPN can detect only hPDPN-expressing cancer cells, not normal cells, including lymphatic endothelial cells and pulmonary type I alveolar cells. Although LpMab-2 might bind to both a peptide and glycans of hPDPN [17], LpMab-23 could detect the conformational change of hPDPN peptides, which might be induced by cancer-specific glycans [38]. Both LpMab-2 and LpMab-23 possess high antitumor activities by those antibody-dependent cellular cytotoxicities (ADCC) [38,39]. Furthermore, LpMab-23-recognizing cancer-type podoplanin could be a novel predictor for a poor prognosis of early stage tongue cancer [40]. Recently, we also utilized a Cell-Based Immunization and Screening (CBIS) method to establish mAbs against various membrane proteins, such as CD133 [41], CD44 [42], PD-L1 [43], pig PDPN [34], horse PDPN [44], and cat PDPN [32]. Importantly, those mAbs are very useful for flow cytometry, Western blot, and immunohistochemistry. In contrast, we could not develop useful mAbs by immunizing synthetic peptides (data not shown). Using selecting one method or the combination of those methods such as CasMab technology and CIBS method, we could produce sensitive and specific mAbs against membrane proteins, which are very useful for not only flow cytometry, but also Western blot and immunohistochemistry when we could not develop mAbs by immunizing synthetic peptides or recombinant proteins.

Indeed, we first tried to produce *anti*-aPDPN mAbs by immunizing synthetic peptides, which are corresponding to PLAG domains of aPDPN; however, we could not obtain any mAbs, which are applicable for Western blot or immunohistochemistry (data not shown). Then, we employed the CBIS method in this study to develop sensitive and specific mAbs against aPDPN for the immunohistochemical analysis of paraffin-embedded tissue sections (Fig. 1). Finally, PMab-225, which is very useful for flow cytometry (Fig. 2), Western blot (Fig. 3), and immunohistochemical analyses (Fig. 4, Fig. 5, Fig. 6), was developed. Interestingly, PMab-225 cross-reacted with human, bovine, tiger, bear, goat, sheep, and whale PDPNs, which were overexpressed in CHO-K1 cells (data not shown), although the percentage of homology of aPDPN with hPDPN is only 66%. In contrast, PMab-225 did not react with mouse, rat, rabbit, dog, cat, pig, Tasmanian devil, and horse PDPNs (data not shown). In future study, we should determine the critical epitope of PMab-225; then, we might uncover the mechanism of cross-reactivity against many species. In immunohistochemical analysis, PMab-225 stained lymphatic endothelial cells (Fig. 5) and pulmonary type I alveolar cells using antigen retrieval with citrate buffer (Fig. 4). However, PMab-225 did not stain alpaca kidney in this condition (data not shown). In contrast, alpaca kidney was stained using antigen retrieval with EnVision FLEX Target Retrieval Solution High pH (Fig. 6). In the future study, we should clarify the molecular difference of aPDPNs, including post-translational modifications in several tissues.

In conclusion, we have established a mAb against aPDPN, PMab-225, which is suitable for use in flow cytometry, Western blotting, and immunohistochemical analyses. PMab-225 should prove useful to elucidate the pathophysiological functions of aPDPN in future studies. In contrast, sensitive and specific mAbs against membrane proteins for alpaca have not been established; therefore, we should develop many mAbs against alpaca membrane proteins, such as CD31 or LYVE-1 for investigation of vascular endothelial cells or lymphatic endothelial cells.

## Conflicts of interest

The authors declare no conflicts of interest involving this article.

## Funding

This research was supported in part by AMED under Grant Numbers: JP18am0101078 (Y.K.), JP18am0301010 (Y.K.), and JP18ae0101028 (Y.K.), and by JSPS KAKENHI Grant Number 17K07299(M.K.K.) and Grant Number 16K10748(Y.K.).
